# Organochlorine pesticide contamination in sediments from Richards Bay, South Africa: spatial trends and ecotoxicological risks

**DOI:** 10.1007/s11356-022-22298-0

**Published:** 2022-08-05

**Authors:** Paul Mehlhorn, Marc Humphries, Julia Gensel, Archibold Buah-Kwofie, Raymond Lubem Tyohemba, Torsten Haberzettl

**Affiliations:** 1 Institute for Geography and Geology, University of Greifswald, F.L.-Jahn Str. 16, 17489 Greifswald, Germany; 2 School of Chemistry, University of the Witwatersrand, Johannesburg, South Africa; 3 MARUM - Center for Marine Environmental Sciences, University of Bremen, Bremen, Germany; 4 Nuclear Power Institute, Ghana Atomic Energy Commission, P. O Box LG 80, Legon, Accra Ghana

**Keywords:** DDT, Distribution, HCH, Malaria, Pollution, Residues, Risk assessment, Stockholm Convention

## Abstract

The occurrence and distribution of organochlorine pesticides (OCPs) in aquatic systems is a matter of global concern and poses significant toxicological threats to both organisms and human health. Despite the extensive use of OCPs for pest and disease control in southern Africa, relatively few studies have examined the occurrence and toxicological risks of OCP residues in the region. This study investigates the composition, distribution, and potential sources of OCP contamination in sediments from Richards Bay, a rapidly developing industrial port on the northeast coast of South Africa. Surface sediments collected from Richards Bay Harbour and surrounding areas indicate that OCP contamination in the region is widespread. Total concentrations (∑OCP) in surface samples ranged from 135 to 1020 ng g^−1^, with hexachlorocyclohexanes (∑HCH; 35–230 ng g^−1^) and dichlorodiphenyltrichloroethanes (∑DDT; 12–350 ng g^−1^) the dominant contaminant groups detected. Metabolite isomeric compositions indicate that the presence of aldrin and endosulfan likely result from historical agricultural usage, while recent input of γ-HCH, heptachlor, and endrin may be linked to the illegal use of old pesticide stockpiles. Total DDT concentrations were dominated by p,p′-DDT (80 ± 64 ng g^−1^), which was attributed to its ongoing use in malaria vector control in the region. A ^210^Pb-dated sediment core revealed that OCP input to the local environment increased dramatically from relatively low concentrations in the mid-1940s (∑OCP, 355 ng g^−1^) to peak levels (∑OCP, 781 ng g^−1^) in the 1980s/1990s. An overall decrease in ∑OCP concentration from the mid-2000s is likely related to restrictions on use following the Stockholm Convention in 2004. Despite current restrictions on use, OCP concentrations exceeded sediment quality guidelines in the vast majority of cases, raising concerns for protected estuarine and mangrove habitats in the area, as well as for local fishing and farming communities.

**Supplementary Information:**

The online version contains supplementary material available at 10.1007/s11356-022-22298-0.

## Introduction

Organochlorine pesticides (OCPs) are of great environmental concern due to their environmental persistence and potential for bioaccumulation (Potapowicz et al. 2020). Once introduced into the environment, various transport processes result in their widespread dispersal and accumulation in different media, with aquatic coastal ecosystems, such as estuaries and coastal lakes, acting as important sinks for agricultural and industrial pollution (Saulnier and Mucci 2000; Mestres et al. 2006). Bioaccumulation (bioconcentration and biomagnification) of OCPs through the food chain can pose ecological and human health risks in local and regional communities (Bornman et al. 2010; Humphries 2013; Gerber et al. 2016; Volschenk et al. 2019; Buah-Kwofie and Humphries 2021).Their bioaccumulation potential, toxicity, and ability for endocrine disruption make these pesticides a matter of global concern (Bouwman 2004; Buah-Kwofie et al. 2018, 2019; Tyohemba et al. 2020).

**Fig. 1:**
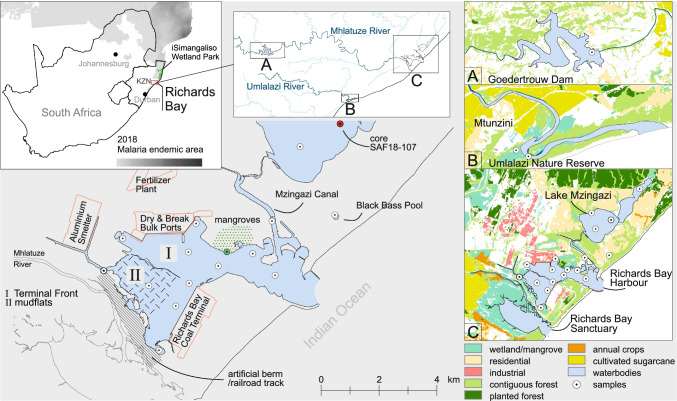
Top left: malaria endemic regions of South Africa (modified from Weiss et al. 2019) KwaZulu-Natal (KZN). Top middle: catchment area of Mhlatuze and Umlalazi Rivers. Right: sampling locations and regional overview of Goedertrouw Dam (A), Umlalazi Nature Reserve (B), and Richards Bay (C) showing dominant land use (modified from Thompson 2019). Note the characteristic change in land use from north-east (residential and industrial) to south-west (wetland/mangrove habitat) of Richards Bay. Middle: Richards Bay Harbour and adjacent industries, as well as the mangrove forest sampling location (green)

South Africa is the largest consumer of pesticide products in Africa and is ranked as the 20th highest user in the world (FAO 2020). The use of OCPs in agriculture and disease vector programs in South Africa dates back to the 1940s (Fischer et al. 2011; Quinn et al. 2011; Buah-Kwofie et al. 2018). Although environmental and human health concerns led to their ban in the early 1970 in most developed countries, OCPs remained widely in use in South Africa until the signing of the Stockholm Convention on Persistent Organic Pollutants (POPs) in 2004 (Bouwman 2004). While the use of most OCP compounds is today banned in South Africa, DDT is still used to control the spread of malaria in the north-eastern parts of the country. The application of DDT by indoor residual spraying (IRS) remains the primary method of control by local health departments, particularly in the province of KwaZulu-Natal (Maharaj et al. 2019) (Fig. 1). As a result, DDT and other legacy OCP compounds continue to be detected in environmentally significant concentrations in the region, with several studies reporting on the widespread prevalence of OCPs in both sediment (Humphries 2013; Buah-Kwofie and Humphries 2017) and wildlife species (Buah-Kwofie et al. 2018; Bouwman et al. 2019; Humphries et al. 2021) from iSimangaliso Wetland Park (Fig. 1).

Apart from the work conducted in iSimangaliso Wetland Park, little is known about the prevalence of OCPs elsewhere along the coastline of KwaZulu-Natal. Early studies carried out by the South African marine pollution survey on sediments and fish at Richards Bay Harbour, located about 60 km south of iSimangaliso (Fig. 1), indicated only low quantities of DDT and dieldrin in samples of fish (Cloete and Oliff 1976). A few years later, the detection of DDT in fish from Richards Bay Harbour suggested recent inputs of technical DDT from local mosquito control activities (Gardner et al. 1983). More recently, high concentrations of hexachlorocyclohexanes (HCHs) in sediments from Richards Bay were reported by Roos (2010); however, the sampling conducted as part of this survey was limited in scope and did not consider sediments found within Richards Bay Harbour itself. Richards Bay Harbour functions as South Africa’s leading coal export facility, around which various other industries and bulk cargo export facilities have developed. Although Richards Bay is one of the fastest developing industrial nodes in the country and host to some of the largest sub-tropical mangrove forests (Fig. 1) in South Africa (Naidoo 2016), a detailed evaluation of OCP contamination in the region is yet to be conducted.

This study aims to examine the concentration and distribution of OCPs in the Richards Bay area. Using a combination of surface sediment and core sampling, we examine spatial and temporal trends in OCP composition and concentrations to identify potential sources and sinks of contaminants. We integrate our findings with results from previous studies and assess the ecotoxicological implications of OCPs in Richards Bay.

## Materials and methods

### Study site

Established in 1976, Richards Bay Harbour has become one of the largest exporting ports in Africa. Prior to port construction, Richards Bay was the direct estuary of the Mhlatuze River. This connection was cut off during port construction to minimize the effect of siltation, separating Richards Bay Harbour from the Mhlatuze River, which today flows into the Richards Bay Sanctuary (Fig. 1). Industries associated with the port of Richards Bay include the Richards Bay Coal Terminal, an aluminum smelter and exporting freight terminals (Terminal Front) (Fig. 1). Freshwater input to Richards Bay Harbour is supplied via the Mzingazi Canal, which connects the port to Lake Mzingazi (Fig. 1c). Black Bass Pool is a small, freshwater lake located within rural residences of Richards Bay and is not hydrologically connected to Lake Mzingazi or Richards Bay Harbour.

### Sample collection

Surface sediment samples were collected during two surveys carried out in 2018 and 2019. Samples from Richards Bay Harbour (*n* = 12), Lake Mzingazi (*n* = 3), Black Bass Pool (*n* = 1), Mangroves at RBH (*n* = 1), Goedertrouw Dam (*n* = 1), and Umlalazi Nature Reserve (*n* = 2) were retrieved using an Ekman-Birge bottom sampler (HYDROBIOS, Kiel, Germany). To examine the most recent sediment deposits, only the topmost 1 cm of the grab was removed with a metal spoon, transferred into a 10-ml glass vial, and kept frozen until further processing (Mehlhorn et al. 2021).

To examine temporal variations in OCP accumulation, a 113-cm sediment core (SAF18-107; Fig. 1) was retrieved from Lake Mzingazi using a modified ETH-gravity corer (Kelts et al. 1986) with 63-mm tube diameter. The core was split longitudinally, photographed, and lithologically described at the Physical Geography Department of the University of Greifswald. Surface sediments and core sub-samples (4-cm intervals, *n* = 4) were subsequently freeze-dried and ground into fine powder using a ball mill for analysis.

The area of Richards Bay is regarded the final sink for sediments originating from within the Mhlatuze catchment area (Fig. 1b). In order to assess the input of contaminants originating from farther inland, the Goedertrouw Dam was sampled. The Umlalazi Nature Reserve (UMR), near the township of Mtunzini, extends the sample range to the next southern catchment. Two sampling sites were selected, at the border of the mangrove forest and within the Umlalazi River, close to a slipway (Fig. 1b).

### Dating

The top 16 cm of sediment core SAF18-107 was radiometric dated using ^210^Pb. Measurements of ^210^Pb were made by gamma ray spectrometry using a high-purity germanium detector system at the Laboratoire de Radiochronologie, Université Laval, Quebec, Canada. To establish a chronology, age-depth modeling was performed following the procedure by Aquino-López et al. (2018), using the R package *rplum* version 0.2.2 (Blaauw et al. 2021).

### TOC measurement

Total organic carbon (TOC) was determined by combustion of dried and homogenized samples with a CNS elemental analyser (EuroEA) at the University of Greifswald. Inorganic carbon was removed from sediments prior to measurement using 20% HCl. Control samples were measured in triplicate and did not exceed an error of 3%.

### OCP extraction and analysis

The extraction of OCPs was carried out at the University of the Witwatersrand following previously validated methods (Buah-Kwofie and Humphries 2017). Briefly, 3–5 g of dried material was rehydrated in water and extracted with 10 ml acetonitrile (containing 1% glacial acetic acid). A mixture of anhydrous magnesium sulfate (MgSO_4_; Sigma-Aldrich), sodium acetate (NaOAc; Sigma-Aldrich), and sodium acetate trihydrate (NaOAc $\bullet$ 3H_2_O; Sigma-Aldrich) was added to aid in the separation of aqueous and organic phases. The sample was centrifuged and an aliquot of the organic phase was cleaned using a combination of MgSO_4_, C18 (Supelco), and primary secondary amine (PSA; Supelco). This mixture was vortexed and then centrifuged to isolate the clean extract. An aliquot of the extract was concentrated to dryness under vacuum at a temperature of < 40 °C, reconstituted in hexane, and spiked with internal standard (pentachloronitrobenzene) for final analysis. The recovery efficiency of this method has been shown to range between 68 and 115% (Buah-Kwofie and Humphries 2017; Table S1).

A total of 17 OCPs were analyzed, which included dichlorodiphenyltrichloroethanes (DDTs; p,p′-DDT, p,p′-DDE, and p,p′-DDD; sum expressed as ∑DDT), hexachlorocyclohexanes (HCHs; α-, β-, γ-, δ-HCH; sum expressed as ∑HCH), drin-residues (aldrin, dieldrin, endrin, and endrin ketone; sum expressed as ∑drins), endosulfans (α-, β-endosulfan, and endosulfan-sulfate; sum expressed as ∑endosulfans), and chlor-residues (heptachlor, heptachlor epoxide, and methoxychlor; sum expressed as ∑chlors). Analysis was performed by two-dimensional gas chromatography‒time-of-flight mass spectrometry (GC X GC-TOFMS) using an Agilent 7890 GC coupled to a Leco Pegasus 4D TOF mass spectrometer, as previously described by Buah-Kwofie and Humphries (2017). Data processing and peak identification were performed using the Leco ChromaTOF software, with peaks identified based on retention time and confirmed by two identifier ions. Quantification was performed using high-purity (> 98%) PESTANAL® reference standards purchased from Sigma-Aldrich. Correlation coefficients derived from linear regressions obtained from matrix-matched calibration curves were > 0.99 in all cases.

Sample extracts were analyzed in duplicate with relative standard deviations typically < 15%. Quality control standards were run after every third sample to monitor and correct for variations in instrument response. Detection limits ranged between 0.42 and 3.1 ng g^−1^ dry weight (dw).

### Data treatment

Descriptive data analysis was performed using R version 4.1.0 (R Core Team 2021). To test for normal distribution of the data, the Shapiro–Wilk test was performed, revealing summed data to be mainly non-parametric (with the exception of ∑HCH and ∑DDT/TOC). Hence, Spearman rank correlation coefficients were calculated using the function "cor.test(…,method = "spearman")", and probability values less than *p* < 0.05 were considered statistically significant. Concentrations below detection limit were assigned a value of zero.

Spatial distribution maps were created using the inverse distance-weighted technique of the Spatial Analyst function in ArcMap 10.6.1.

## Results and discussion

### OCP concentrations and spatial distributions

OCPs were detected at all sampling sites with total concentrations (∑OCP) ranging between 135 and 1020 ng g^−1^ (Table 1). All target residues were detected in the vast majority of samples analyzed. ∑HCH varied from 35 to 230 ng g^−1^, ∑DDT from 12 to 350 ng g^−1^, ∑drins from 43 to 270 ng g^−1^, ∑endosulfans from 21 to 260 ng g^−1^, and ∑chlor from 18 to 280 ng g^−1^.

**Table 1:** Mean concentrations ± SD and (min − max) of OCP metabolites in surface sediment samples from Richards Bay (ng g^−1^ dw)

Analytes	Concentration (ng g^−1^) dw					
	Richards Bay Harbour (*n* = 12)	Lake Mzingazi (*n* = 3)	Black Bass Pool (*n* = 1)	Mangroves at RBH (*n* = 1)	Goedertrouw Dam (*n* = 1)	Umlalazi Nature Reserve (*n* = 2)
α-HCH	27 ± 14 (11–53)	25 ± 5.7 (18–32)	27	26	22	9.5 (8.0–11)
β-HCH	33 ± 14 (12 − 58)	46 ± 24 (18 − 76)	76	31	24	14 (9.0 − 18)
δ-HCH	14 ± 5.9 (7.0 − 28)	37 ± 20 (12 − 60)	58	20	13	11 (7.0 − 14)
γ-HCH	48 ± 34 (10 − 110)	37 ± 6.8 (32 − 47)	35	17	47	8.5 (7.0 − 10)
**∑HCHs**	**120 ± 63 (42 − 230)**	**150 ± 55 (81 − 220)**	**200**	**94**	**110**	**43 (35 − 50)**
Aldrin	10 ± 3.3 (6.0 − 15)	3.0 ± 4.2 (n.d. − 9.0)	9	18	12	6.5 (4.0 − 9.0)
Dieldrin	16 ± 3.8 (8.0 − 22)	32 ± 14 (13 − 48)	59	22	15	11 (4.0 − 18)
Endrin	18 ± 4.8 (11 − 27)	51 ± 26 (16 − 80)	26	18	18	18 (10 − 25)
Endrin aldehyde	16 ± 8.0 (1.0 − 27)	17 ± 7.0 (8.0 − 25)	30	14	15	9.0 (8.0 − 10)
Endrin ketone	80 ± 66 (3.0 − 190)	10 ± 15 (n.d. − 31)	n.d	9	36	0.5 (n.d. − 1.0)
**∑drins**	**130 ± 74 (49 − 270)**	**110 ± 31 (77 − 150)**	**120**	**81**	**96**	**44 (43 − 45)**
Heptachlor	17 ± 6.6 (4.0 − 31)	35 ± 17 (15 − 56)	n.d	10	21	8.0 (3.0 − 13)
Heptachlor epoxide	10 ± 3.3 (5.0 − 17)	22 ± 10 (8.0 − 33)	25	15	9	6.0 (5.0 − 7.0)
Methoxychlor	19 ± 8.1 (5.0 − 34)	42 ± 50 (n.d. − 110)	250	10	21	6.5 (n.d. − 13)
**∑chlors**	**44 ± 17 (18 − 82)**	**99 ± 56 (37 − 170)**	**280**	**35**	**50**	**20 (18 − 23)**
p,p′-DDE	7.9 ± 3.0 (4.0 − 13)	15 ± 6.2 (6.0 − 20)	83	11	7	5.5 (3.0 − 8.0)
p,p′-DDD	13 ± 4.8 (3.0 − 19)	13 ± 4.5 (8.0 − 19)	53	10	7	5.0 (4.0 − 6.0)
p,p′-DDT	48 ± 51 (4.0 − 200)	53 ± 59 (n.d. − 140)	220	17	77	23 (n.d. − 45)
**∑DDTs**	**69 ± 51 (15 − 210)**	**81 ± 65 (31 − 170)**	**350**	**38**	**92**	**33 (12 − 54)**
α-Endosulfan	12 ± 3.0 (7.7 − 19)	24 ± 11 (9.6 − 35)	45	15	12	6.8 (5.2 − 8.4)
β-Endosulfan	18 ± 7.4 (8.6 − 33)	13 ± 7.2 (6.3 − 23)	17	14	15	6.2 (6.1 − 6.4)
Endosulfan sulfate	70 ± 70 (7.3 − 220)	22 ± 5.4 (17 − 30)	11	13	31	7.7 (6.4 − 9.1)
**∑endosulfans**	**100 ± 75 (29 − 260)**	**60 ± 21 (37 − 87)**	**72**	**42**	**58**	**21 (21 − 21)**
**∑OCP**	**470 ± 210 (150 − 830)**	**500 ± 180 (270 − 720)**	**1020**	**290**	**400**	**160 (140 − 190)**

Rounded to two significant figures. *n.d.* not detected

Metabolite sums are highlighted in bold

**Fig. 2:**
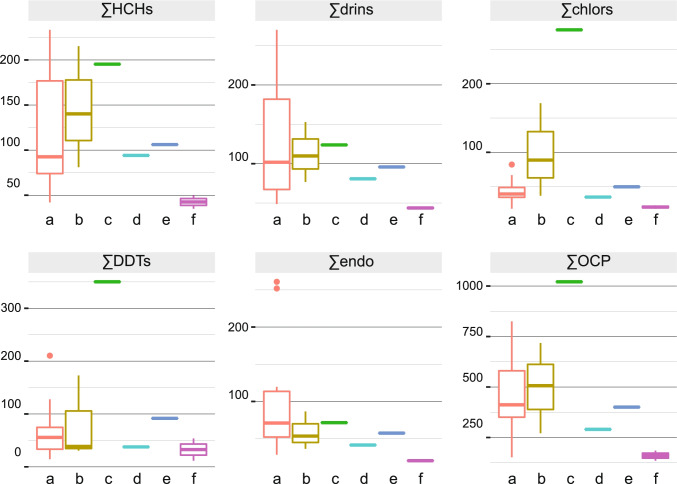
Summed analyte classes and total OCP concentration (∑OCP) ordered by sampling location: a Richards Bay Harbour, b Lake Mzingazi, c Black Bass Pool, d Mangroves at Richards Bay Harbour, e Goedertrouw Dam, and f Umlalazi Nature Reserve. The whiskers of the box plot indicate the 1.5 IQR, and dots indicate outliers. See Fig. 1 for locations of sampling sites

The average ∑OCP concentration in Richards Bay Harbour was 470 ± 210 ng g^−1^ and was similar to total concentrations measured in Lake Mzingazi (500 ± 180 ng g^−1^) and at Goedertrouw Dam (400 ng g^−1^) (Fig. 2). Samples from the Umlalazi Nature Reserve were characterized by overall lower ∑OCP concentrations (160 ng g^−1^). Sediment from the mangroves at Richards Bay Harbour indicated intermediate amounts of OCPs (290 ng g^−1^), while Black Bass Pool exhibited the highest ∑OCP concentration (1020 ng g^−1^).

### DDT

**Fig. 3:**
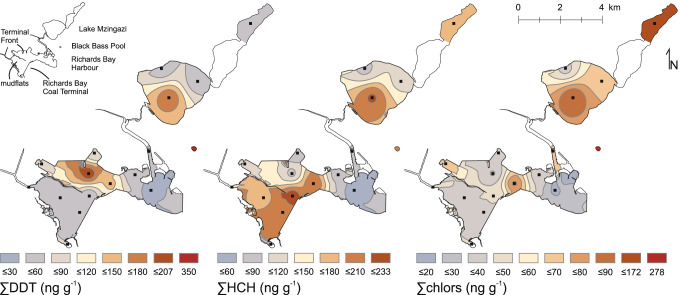
Spatial distribution maps of the metabolite sums ∑DDT, ∑HCH, and ∑chlors (left to right)

∑DDT concentration in surface sediments from Richards Bay Harbour varied from 15 to 210 ng g^−1^ (Fig. 2). Highest concentrations occurred at the Terminal Front (Fig. 3), with a general decline towards the Indian Ocean. Sediments from Lake Mzingazi were characterized by similar ∑DDT concentrations, ranging between 31 and 170 ng g^−1^. Black Bass Pool revealed the highest ∑DDT values at 350 ng g^−1^, while substantially lower concentrations were detected in river bed (54 ng g^−1^) and mangrove (12 ng g^−1^) sediments from Umlalazi Nature Reserve, and in sediment from Goedertrouw Dam (92 ng g^−1^).

### HCH

∑HCH concentrations in surface sediments from Richards Bay Harbour ranged from 42 to 230 ng g^−1^, with highest concentrations detected near the Coal Terminal (Fig. 3). ∑HCH in surface sediments from Lake Mzingazi varied from 81 to 215 ng g^−1^ and showed a similar distribution to ∑DDT. Total HCH concentrations at Black Bass Pool (195 ng g^−1^) were similar to maximum concentrations in Richards Bay Harbour and Lake Mzingazi, while samples from Umlalazi indicated lower concentrations (35–50 ng g^−1^). The inland location at Goedertrouw Dam indicated an intermediate concentration of 110 ng g^−1^ (Fig. 2).

### Other OCP residues

The distribution of chlor residues (Fig. 3) in Richards Bay Harbour was confined to three local maxima: mid-port, at the north-western bulk-terminal, and inside the Mzingazi Canal. In Lake Mzingazi, high concentrations were found in the center of the main lake (90 ng g^−1^) and the north-eastern lake (170 ng g^−1^). Highest chlor concentrations were found at Black Bass Pool (280 ng g^−1^), while relatively low levels were detected at Umlalazi Nature Reserve (avg. 20 ng g^−1^) and Goedertrouw Dam (50 ng g^−1^).

Concentrations of ∑drins and ∑endosulfans in Richards Bay varied from 49 to 270 ng g^−1^ and 29 to 260 ng g^−1^, respectively. In general, ∑drins, ∑endosulfans, and ∑OCP showed similar distribution patterns to ∑HCHs (Fig. 3), as indicated by correlation factors of 0.9 (*p* < 0.001), 0.81 (*p* < 0.001), and 0.95 (*p* < 0.001), respectively.

### Temporal variations in OCP accumulation

**Fig. 4:**
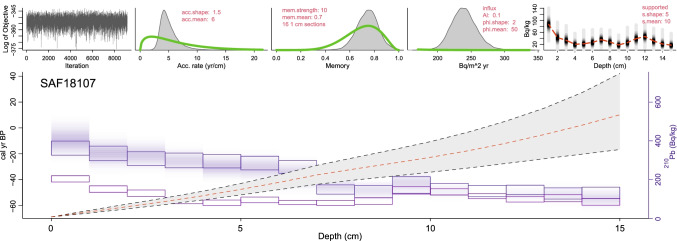
The.^210^Pb-derived age-depth model of sediment core SAF18-107 using the Bayesian chronological approach of Aquino-López et al. (2018)

**Fig. 5:**
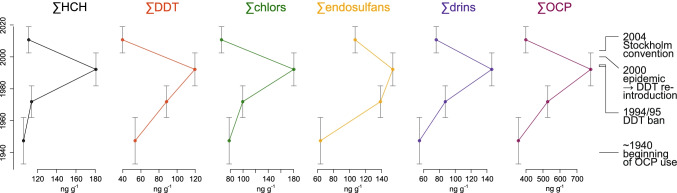
Concentrations of OCP metabolites over time from sediment core SAF18-107 recovered from Richards Bay Harbour. Historical events are modified from Coetzee et al. (2013)

The ^210^Pb-derived age model for the top 15 cm of core SAF18-107 provided a chronology for the past ~ 85 ± 30 years (Fig. 4; Table S2). The concentration of ∑OCPs steadily increases from 355 ng g^−1^ in 1947 (± 15 years) to 781 ng g^−1^ in 1992 (± 10 years), and then decreases to 400 ng g^−1^ in the most recent sample (2011 ± 8 years) (Fig. 5). Total concentrations measured of individual metabolite sums indicate very similar trends (Fig. 5) and correspond well with the rise in pesticide usage in South Africa during the mid-1940s to mid-1990s. The peak in pesticide concentrations from 1982 to 2003 covers a time period where OCPs were used extensively in agriculture and for disease vector control. Observed decreases in OCP concentration over the last two decades correspond with government actions limiting the use of OCPs in the 1990s and eventual ban under the Stockholm Convention in 2004. Although use of DDT was reintroduced in 2000 and continues to be employed today, application is implemented in a more targeted manner through IRS.

### OCP compositions and potential sources

**Fig. 6:**
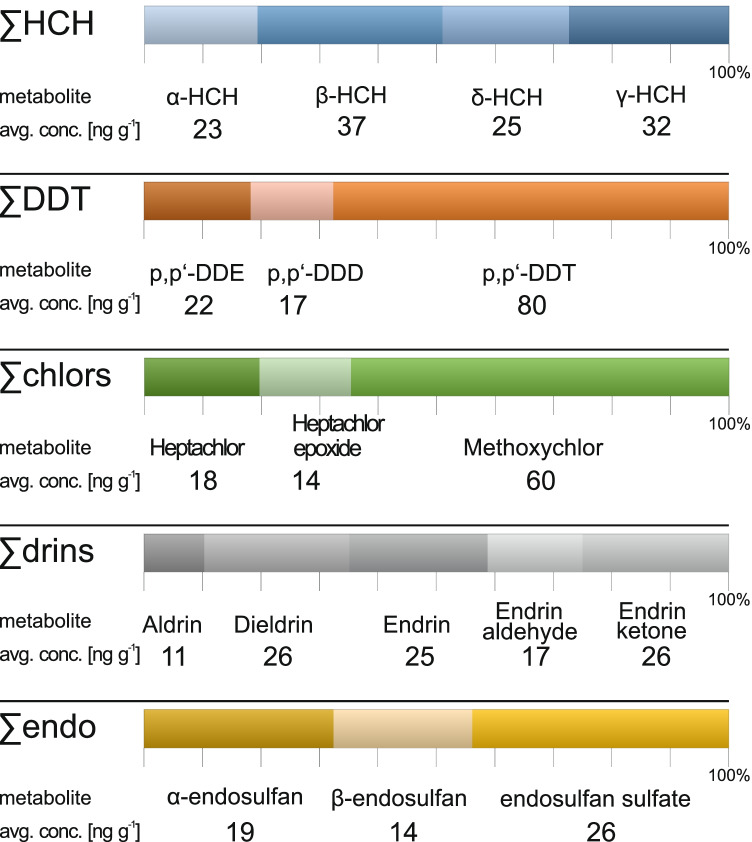
Average composition and concentration of individual metabolite in surface sediments from the Richards Bay region

On average, ∑DDT concentrations in Richards Bay surface sediment samples were composed of 68% p,p′-DDT, 14% p,p′-DDD, and 18% p,p′-DDE (Fig. 6). Technical DDT consists of ~ 77% p,p′-DDT, 15% o,p′-DDT, and negligible percentages of its metabolites p,p′-DDD (0.3%) and p,p′-DDE (4%) (World Health Organization (WHO) 1989). The relatively high proportion of p,p′-DDT measured in samples thus suggests recent input of technical DDT into the environment at Richards Bay.

The ratio between the parent compound and the sum of its metabolites ($\frac{\mathrm{p},{\mathrm{p}}^{\mathrm{^{\prime}}}-\mathrm{DDT}}{\mathrm{p},{\mathrm{p}}^{\mathrm{^{\prime}}}-\mathrm{DDD }+\mathrm{ p},{\mathrm{p}}^{\mathrm{^{\prime}}}-\mathrm{DDE}}$) can be used to distinguish between current and historical usage of DDT (Qian et al. 2006). Over time, the proportion of metabolites is expected to increase, with relatively low DDT/(DDD + DDE) ratios (i.e., < 1) being indicative of historical inputs. Hiller et al. (2011) used a ratio of 0.4 to distinguish between recent and historical DDT inputs. Sediments from Richards Bay Harbour and surrounding environments are characterized by DDT/(DDD + DDE) ratios that greatly exceed 1 (mean: 2.7), suggesting the recent use of DDT as part of ongoing malaria vector control through IRS. In contrast, very low DDT ratios (< 0.4) were reported for iSimangaliso Wetland Park (Humphries 2013; Buah-Kwofie and Humphries 2017), indicating relatively long-term degradation. The comparably high DDT ratios measured in sediments from Richards Bay likely reflect a close proximity to IRS-treated areas.

Lindane (pure γ-HCH) has a half-life of about 2 weeks, and is degraded by microorganisms (Benezet and Matsumura 1973) and photo-chemically isomerized to α-HCH (Malaiyandi and Shah 1984). The α- and γ-isomers degrade to the more stable β-form (Walker et al. 1999; Willett et al. 1998), which is highly persistent in the environment (Malik et al. 2007). In contrast, technical HCH usually contains only 10–12% γ-HCH (Saadati et al. 2012). At Richards Bay, γ-HCH comprises an average of 27% of ∑HCH (Fig. 6), suggesting the recent input of lindane at all sites. This is in contrast to sites situated in northern KwaZulu-Natal where γ-HCH was rarely detected in sediments from Lake Sibaya and Kosi Bay (Buah-Kwofie and Humphries 2017).

Aldrin was present in lower abundance compared to dieldrin (aldrin/dieldrin ratio = 0.6; Fig. 6), which is attributed to the long-term degradation of aldrin to the more stable and persistent dieldrin metabolite. This is similar to what Buah-Kwofie and Humphries (2017) reported in sediments from iSimangaliso Wetland Park and what Gerber et al. (2015) reported in river sediments from Kruger National Park.

Although endrin has a half-life of 12–20 years and was banned in South Africa during the 1980s (Fischer et al. 2011), the detection of relatively high proportions of endrin relative to its metabolites (endrin ketone and endrin aldehyde) in surface sediment samples suggest possible recent contamination/use. In sediment, the rapid metabolization of heptachlor (half-life of 1–3.5 days; Reed and Koshlukova 2014) to the more stable heptachlor epoxide is expected (World Health Organization (WHO) 2006). However, at most sampling sites the heptachlor/heptachlor-epoxide ratio exceeded 1, indicating potential recent inputs in Richards Bay. This contrasts with northern sites in iSimangaliso Wetland Park (Buah-Kwofie and Humphries 2017) and surrounding river catchments (Buah-Kwofie and Humphries 2021) which indicate historical use of heptachlor.

The β-endosulfan degrades slower than α-endosulfan towards the more stable endosulfan-sulfate (Ghadiri and Rose 2001). Sediments from Richards Bay were characterized by average α-endosulfan/β-endosulfan metabolite ratios of < 1 and suggest that historic input corresponds to sites in iSimangaliso Wetland Park (Buah-Kwofie and Humphries 2017).

### Sources and sinks of contamination

OCPs bind strongly to organic matter, which can affect their representation in heterogeneously composed surface samples. In order to reduce matrix effects and identify potential sources of contamination, OCP concentrations were normalized to TOC.

**Fig. 7:**
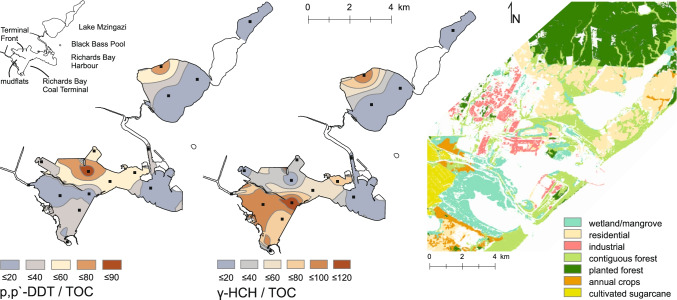
Spatial distribution patterns of p,p′-DDT/TOC (left), y-HCH/TOC (middle), and land use data (right; modified from Thompson 2019). To account for matrix effects, isomeric metabolite concentrations are normalized to TOC. At Lake Mzingazi elevated ratios occur in the northwest of the lake. In Richards Bay Harbour, increased p,p′-DDT ratios locate at the Terminal Front, in conjunction with the main industrial site of the port. y-HCH ratios locate at the mudflats and the Coal Terminal where effluents of nearby farmland might drain into the port via the canal systems

The distribution map of p,p′-DDT/TOC shows elevated ratios inside the industrial port section of Richards Bay Harbour and along the western margin of Lake Mzingazi (Fig. 7). Extensive development (residential and industrial; Fig. 7) associated with the town of Richards Bay is a likely source for DDT entry (i.e., by IRS).

In contrast, high γ-HCH/TOC ratios are found in front of the Richards Bay Coal Terminal and extend into the mudflats, a non-dredged, shallow water area of the port that experiences little marine traffic (Fig. 1; Fig. 7). Farmland directly west of Richards Bay Harbour, including commercial sugar cane cultivation and subsistence farming, is a likely source of γ-HCH contamination, which enters the port via nearby canals on the western side of the mudflats.

In Lake Mzingazi, highest normalized p,p′-DDT and γ-HCH values are found along the north western margin of the lake. The adjacent land use is characterized by densely populated residential areas and commercial forestry to the north of Lake Mzingazi (Fig. 7), which is the likely origin of these pollutants.

The TOC-normalized distribution patterns of heptachlor (*r* = 0.81), endrin (*r* = 0.79), aldrin (*r* = 0.78), and α-endosulfan (*r* = 0.82) correlated well with γ-HCH, which indicates a similar source to HCH (i.e., agriculture and forestry).

### Ecotoxicological evaluation

OCPs in sediment have the potential for biological uptake. To evaluate the potential toxicological effects, sediment quality guidelines (SQG) proposed by National Oceanic and Atmospheric Administration (NOAA) (MacDonald et al. 1996, 2000; Long et al. 1995) were employed. Using this approach, each chemical is classified by two guideline values, the effect range low (ERL) which represents the concentration below which adverse effects are rarely observed, and the effect range medium (ERM), above which adverse toxicological effects would frequently occur. Moderate toxicological incidence is expected at concentrations between the ERL and ERM values (Birch and Hutson 2009).

**Fig. 8:**
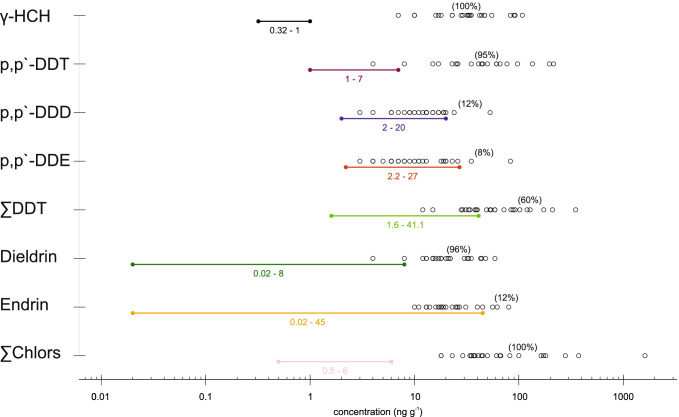
Comparison of individual OCP concentrations (circles) measured in surface sediments from Richards Bay, with sediment quality guidelines proposed by NOAA (Long et al. 1995). Effect Range Low (ERL) and Effect Range Median (ERM) are depicted by colored dots connected by a line. Percentages are given for samples exceeding the ERM

In this study, the ERL threshold is exceeded at all sampling points (Fig. 8). Additionally, all samples (100%) exceeded the γ-HCH and ∑Chlors ERM values, while most (> 90%) also exceeded the p,p′-DDT and dieldrin ERM values (Fig. 8). Therefore, the majority of the samples also indicate a high probability of adverse toxicological effects.

The OCP concentrations measured in sediments from Richards Bay raise ecological and human health concerns. As shown in studies from iSimangaliso Wetland Park and nearby Phongolo River floodplain, OCPs readily enter the food chain and bioaccumulate in aquatic species (Bouwman et al. 2019; Volschenk et al. 2019; Buah-Kwofie and Humphries 2021; Humphries et al. 2021). Potential human health risks may thus exist to local communities who catch fish from the harbour and lakes in Richards Bay or who cultivate crops within the area, as several other studies from the region have demonstrated (e.g., Buah-Kwofie et al. 2019; Volschenk et al. 2019). Additional studies examining bioaccumulation and human health exposure to OCPs at Richards Bay are therefore recommended.

### Regional comparison

**Fig. 9:**
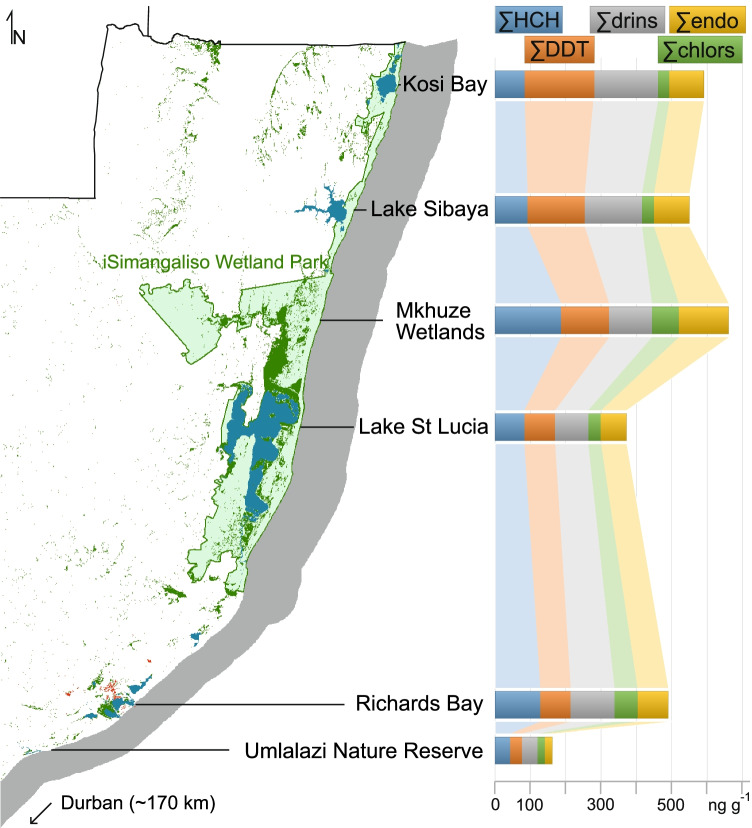
Regional overview of South Africa’s east coast, modified from SANLC 2018 (Thompson 2019) and additionally indicating locations of previous OCP investigations by Buah-Kwofie and Humphries (2017). In side-to-side comparison, locations match the concentrations of OCPs measured in that area. The graph indicates the effect of increased use of pesticides in Richards Bay and the Mkhuze Wetlands. Note general decreasing DDT concentrations from north to south, whereby Lake St. Lucia and Richards Bay indicate similar amounts, despite the regional difference

The sediment OCP concentrations measured at Richards Bay fall within the range previously reported for other coastal systems in northern KZN (Buah-Kwofie and Humphries 2017; Fig. 9). While comparisons between different systems are potentially complicated by variances in the time of sampling, nature of the sediment analyzed, and the sampling strategy employed, regional trends in concentration are evident. In general, catchment size and proximity to potential sources of contamination are important factors influencing total OCP concentrations. Systems associated with large catchment areas impacted by agriculture (e.g., the Mkhuze wetlands) typically show higher levels of OCP accumulation than sites that receive limited amounts of catchment runoff (e.g., Kosi Bay and Lake Sibaya). Total OCP concentrations at Richards Bay are similar to those reported for Kosi Bay and Lake Sibaya, likely indicative of the relatively confined catchment area that characterizes the modern-day system.

Buah-Kwofie and Humphries (2017) noted a clear north to south decreasing gradient in ∑DDT concentrations, which was attributed to IRS and the extent to which DDT is used within the respective catchment areas. St. Lucia and Mkhuze are considered relatively low-risk malaria areas, while outbreaks of malaria have historically been more common in areas farther north. However, DDT concentrations at Richards Bay do not follow this trend, which we attribute to the close proximity of contamination sources (IRS) at this site. ∑DDT concentrations in iSimangaliso Wetland Park are composed predominantly of the metabolites p,p′-DDD and p,p′-DDE. In contrast, p,p′-DDT is the major (80%) DDT metabolite at Richards Bay, indicating that total DDT concentrations at this site are strongly influenced by current IRS practices. Overall, OCP concentrations measured in northern KZN and at Richards Bay contrast strongly with levels measured in the densely urbanized region of Durban, located ~ 170 km south of the present study area (see Fig. 1 and Fig. 9). Vogt et al. (2018) found relatively low ∑endo (0.8 ± 6.0 ng g^−1^), ∑chlor (1.1 ± 3.1 ng g^−1^), and ∑DDT (18 ± 71 ng g^−1^) concentrations at the river and estuary sediments they analyzed from the Durban region, reflecting significantly lower inputs from agricultural and IRS sources.

## Conclusions

This study shows that harbour sediments can be an important sink for organic contaminants. OCPs were widely found in sediments from Richards Bay, the distribution of which could be linked to land use and anthropogenic activities in the region. Agriculture was identified as an important source of legacy OCP contamination, although the recent use of lindane, heptachlor, and endrin, possibly from old stockpiles, is suspected. The prevalence of DDT in sediments was linked to ongoing malaria control measures, with highest concentrations found at sites adjacent to densely populated residential areas. Decreases in OCP concentration over the past two decades indicate the effectiveness of restrictions in reducing contaminant input to the environment. However, despite current restrictions, many of the OCP concentrations measured exceeded sediment quality guidelines and are therefore likely to have adverse impacts on both human and ecosystem health. Further, metabolite ratios suggest that lindane and endrin continue to be used in the region despite current restrictions. The accumulation of substantial quantities of OCP residues within Richards Bay Harbour indicates the need for further monitoring and this study provides important baseline data for future assessments. Furthermore, a detailed investigation into the bioaccumulation of OCPs and associated toxicological risks to biological ecosystems and human health is urgently required.

## Supplementary Information

Below is the link to the electronic supplementary material.

Supplementary file1 (XLSX 10 KB)

Supplementary file2 (XLSX 16 KB)

## Data Availability

The datasets used or analyzed during the current study are available from the corresponding author on reasonable request.
